# A clinical and nonclinical professionals assessment on the perceptions of ISO standards on healthcare practices and patient safety in Saudi Arabia

**DOI:** 10.1097/MD.0000000000043392

**Published:** 2026-02-13

**Authors:** Eyad Talal Attar

**Affiliations:** a Department of Electrical and Computer Engineering, Faculty of Engineering, King Abdulaziz University, Jeddah, Saudi Arabia.

**Keywords:** clinical testing, healthcare practices, healthcare professionals, ISO standards, medical device management, nonclinical adoption, patient safety, Saudi Arabia

## Abstract

International Organization for Standardization (ISO) standards in healthcare aim to improve the quality, safety, and efficiency of medical devices, clinical processes, and health management systems of various countries across the world. This study reviews the perception of healthcare professionals about ISO standards at Saudi Arabia concerning patient safety, medical device management, and their overall influence on clinical and nonclinical practices. The previous study was based on the global healthcare systems of the US and Europe-like developed countries. The online cross-sectional survey of 300 respondents was selected from all regions and professions. Most of the respondents were from urban regions: Riyadh represented 30.4%, Jeddah 25.2%, and the Eastern Province represented 18.6%. The biomedical engineers constituted 43.3%, physicians comprised 29.2%, and most of the participants were males, 87%. Response to the question about ISO standards was that 79.8% agreed that ISO standards play a critical role in improving patient safety; biomedical engineers demonstrated 85.2%, while physicians were at 82.6%. Medical device calibration garnered 81.0% agree that ISO standards on calibration of medical devices are important. Differences across the 4 major regions demonstrated a strong level of agreement among respondents in Riyadh and Makkah but weaker levels among those in Asir and the Northern Borders, as follows: Riyadh 81.5%, Makkah 78.9%, Asir 58.9%, and Northern Borders 53.2%. ISO adoption barriers were reported for mostly nonclinical areas; training shortage, 56.8%, and resource unavailability were the main factors that acted as the main barriers. Besides, full agreement with relevance among ISO standards came to 85.9% among professionals with more than 15 years of experience. The inference to be drawn from such findings is that the same throw a critical light on the reception and application of ISO standards in Saudi healthcare and pinpoints those areas that require further amelioration and awareness across regions and professional groups.

## 1. Introduction

In fact, transformation has taken place within the healthcare sector in recent years as efforts are made to enhance patient safety, improve clinical practices, and modernize healthcare management systems.

This includes high-quality health services in line with the Kingdom-wide Vision 2030, which necessitates adherence to international standards as set out by the International Organization for Standardization (ISO). The healthcare industry has benefited from the implementation of numerous ISO standards that have been developed to enhance the quality, safety, and efficiency of medical devices, clinical procedures, and healthcare management systems worldwide.^[1]^ Over the years, their utility has been vital to establishing a standardized and sustainable means to preserve healthcare quality at regular when keeping patient safety into due consideration.^[2]^ ISO standards have been widely adopted by the Saudi Arabia healthcare system, which is one of the most diverse and fastest-growing in Riyadh, Makkah, Eastern Province, to name a few. This is especially true in medical device administration, clinical testing, and healthcare risk management, where adherence to ISO standards assures both the reliability of health diagnostics and the safe implementation of clinical procedures.^[3]^ However, ISO 9001 and other ISO quality management system standards suffer from limited adoption in the same fields, so this could be due to a lack of awareness about the domains where these standards could be implemented.^[4]^

ISO standards are recognized for ensuring patient safety,^[5]^ supporting doctors and healthcare professionals,^[5]^ and maintaining safety and effectiveness in healthcare through quality and risk management.^[6]^ Additionally, ISO contributes to the evaluation of medical devices,^[7]^ assists in the calibration of medical equipment,^[8]^ and ensures effective sterilization processes and related product quality.^[9]^ The standards also focus on developing consistent terminology and methods for managing safety issues,^[10]^ standardizing practices in oral healthcare and dentistry,^[11]^ ensuring the safety of surgical implants,^[12]^ and contributing to the safe disposal of medical devices.^[13]^ Furthermore, ISO supports nonclinical healthcare operations^[14]^ and promotes compatibility and interoperability in health data and systems.^[15]^ Table 1 compares previous studies with the proposed Study.

**Table 1:** Comparison of previous studies with proposed study.

Aspect	Previous studies	Current study (Saudi Arabia)
Focus area	Primarily focused on global healthcare systems in developed countries like the U.S. and Europe.^[16–18]^	Focused on the Saudi Arabia healthcare system, including both urban and rural regions.
Patient safety	ISO standards significantly enhance patient safety by reducing errors and ensuring procedural consistency.^[16–18]^	79.8% of respondents in Saudi Arabia agreed ISO standards play a critical role in enhancing patient safety.
Medical device calibration	Emphasis on the importance of ISO standards for ensuring accurate calibration of medical devices.^[19,20]^	81.0% of Saudi respondents recognized the critical role of ISO standards in ensuring device accuracy and calibration.
Adoption in nonclinical areas	Limited research on the adoption of ISO standards in nonclinical settings, such as administration and logistics.^[21]^	Lower awareness (63.4%) of ISO standards in nonclinical settings, indicating implementation challenges.
Professional differences in perception	Previous studies noted gaps in awareness among nontechnical healthcare staff.^[21]^	Significant gaps between technical (biomedical engineers, physicians) and nontechnical staff (administrators, nurses).
Regional differences	Focus on developed countries with uniform healthcare systems.	Regional disparities: urban areas like Riyadh and Jeddah showed higher ISO adoption, while rural regions lagged behind.
Age and experience impact	Not a primary focus of previous studies.	Younger professionals in Saudi Arabia, especially in cutting-edge hospitals, showed more adaptability to ISO standards.
Barriers to implementation	Lack of resources and resistance to change are noted in nonclinical settings.^[21]^	Similar barriers were found, with the addition of training deficits, particularly in rural areas and nonclinical roles.
Future recommendations	Broaden ISO standard education across all professional roles and healthcare systems.^[16–18]^	It also targets the training, especially within nonclinical areas, and regional imbalances.

This study finds out the perception of health professionals in Saudi Arabia concerning the impact ISO standards have had on patient safety, management of medical devices, and health practices. Responses from the questionnaire completed by biomedical engineers, physicians, nurses, and healthcare administrators give an overall picture of the opinions and experiences of persons working in the healthcare system. These references thus allow the study to develop 14 key questions used in the survey and present insight into the strengths and limitations of the implementation of ISO standards across different regions and professions.

It helps in understanding how such standards are perceived and applied within the context of the Saudi healthcare system. As ISO standards are at the center in ensuring that health services provision is safe and effective, perceived effectiveness contextualizes the detailed nuts and bolts of what health professionals genuinely expect or think about their expectations. In light of this information, the present study contributes to the ongoing debate about the relevance and adaptability of ISO standards within Saudi Arabia healthcare sector, which highlights certain areas where improvement can be made and strategies through which implementation at both clinical and nonclinical levels can be enhanced.

## 2. Methods

### 2.1. Study design

The following cross-sectional survey design research instrument has been forwarded to healthcare professionals working in various regions of Saudi Arabia in order to seek perceptions. This survey has been designed to capture the perception related to ISO standards in healthcare regarding patient safety, management of medical devices, and the consequences of such standards on clinical and nonclinical practices. Therefore, both closed and open-ended questions were combined to allow the collection of quantitative and qualitative data.

### 2.2. Survey structure

The survey was structured into several key sections:

- Demographics: information regarding gender, age, professional role, and region of practice.
- Perceived relevance of ISO Standards: questions probed into understanding and perceived importance of the ISO standards for clinical operations, patient safety, calibration of medical devices, and applications outside of clinical use.
- Challenges/Barriers: Open-ended questions regarding challenges perceived by healthcare professionals in implementing ISO standards.

The questionnaire was designed with 14 key questions, covering various aspects of healthcare, ensuring a comprehensive evaluation of ISO standard perceptions across different professions and regions.

### 2.3. Sampling and participants

All the workers in healthcare, whether clinically or non-clinically, were targeted: biomedical engineers, physicians, nurses, and healthcare administrators. A convenience sampling method was employed whereby questionnaires were forwarded through institutions of healthcare and professional networks in Riyadh, Makkah, Eastern Province, Asir, and Northern Borders of Saudi Arabia.

A goal was to obtain 600 respondents, but due to some restrictions, 300 were actually gathered. The sample is a wide population of professionals at different levels of the healthcare system.

### 2.4. Data collection

The data collection took 3 months, from January 2023 up until March 2023. Electronic questionnaires will be shared via individual emails and popular social media platforms among health professionals. Web-based data collection was used to ensure the anonymity and confidentiality of respondents. Participation in this study was based on a voluntary response, with informed consent sought for every respondent before filling in the survey.

### 2.5. Data analysis

Quantitative data were analyzed using descriptive statistics to summarize the survey findings. Proportions and percentages were calculated to represent healthcare professionals’ overall perceptions of ISO standards. Cross-tabulations were performed to compare responses between different professional groups and regions. Qualitative data were analyzed using thematic analysis to identify themes and challenges related to the implementation of ISO standards. Coding and categorization of responses from open-ended questions identified the patterns of challenges and facilitators perceived by healthcare professionals.

### 2.6. Statistical tools

Python was used for statistical analysis. Frequencies and percentages were calculated as descriptive statistics for demographic variables and closed-ended questions. ANOVA tests were conducted to compare significant differences in perceptions across different ages and professional years of experience. The *P*-value considered for statistical significance was *P* < .05.

### 2.7. Ethical considerations

Ethical approval for this study was obtained from the Institutional Review Board at one of the leading Saudi hospitals. It was stated that participation in this study was wholly on a voluntary basis and all participants were informed about the purpose of the research. Thereafter, confidentiality was strictly upheld whereby no personal identifiable information was therefore collected in the process of doing the survey. The study was further approved by the Ethical Approval Committee at the Department of Electrical and Computer Engineering, King Abdulaziz University, with Approval No. EAC-02-01-06-23.

## 3. Results

This was a cross-sectional survey study to assess healthcare professionals’ perceptions about ISO standards across Saudi Arabia. The questionnaires encompassed closed and open-ended questions regarding patient safety and medical device management, or the broader impacts associated with ISO standards on clinical and nonclinical practices. Their sample included 500 healthcare professionals from different professions and regions. These contributed to some valuable insight into how ISO standards are accepted and implemented.

Responses showed that the greater number of participants hailed from urban areas: 30.4% were from Riyadh, 25.2% from Jeddah, and 18.6% from the Eastern Province. Biomedical engineers were the greater part of the population with 43.3%, while physicians comprised 29.2% of the population. The gender distribution was highly male-dominated, with 87% of males participating and only 13% of females participating.

A total of 79.8% strongly agreed that the ISO standards played a critical role in enhancing patient safety. The biomedical engineers and physicians were highly in agreement at 85.2% and 82.6%, respectively (*P* < .05). For calibration of the medical devices 81.0% agreed that the ISO standards provided a useful contribution, while for biomedical engineers, it was 89.3%. These findings are supported by international studies that show how ISO standards minimize medical errors and improve procedural consistency.

The analysis revealed regional differences in perceptions of ISO standards, with respondents from Riyadh (81.5%) and Makkah (78.9%) showing stronger agreement compared to those in regions like Asir (58.9%) and the Northern Borders (53.2%) (Table 2). Profession-based differences were also evident, as healthcare administrators (60.5%) and nurses (68.1%) showed lower levels of awareness and agreement compared to biomedical engineers and physicians.

**Table 2:** Agreement on ISO standards by region.

Region	Agreement on ISO standards (%)
Riyadh	81.5
Makkah	78.9
Eastern Province	77.3
Asir	58.9
Northern borders	53.2

ISO = International Organization for Standardization.

Several obstacles to compliance with ISO standards were mentioned in the survey, especially outside of nonclinical settings. These numbers were even higher in rural areas, and the biggest obstacles to saving lives were said to be: lack of training (56.8%) and inadequate resources (48.5%). Agreement with the question of whether ISO standards were adhered to in nonclinical roles only reached 63.4% overall, with compliance perceived to be lowest among healthcare administrators at 55.8%. Table 3 shows the agreement on the 14 questions.

**Table 3:** Summary of results from ISO standard survey.

ISO-related question	Response options	Percentage of respondents	
1. Do you agree that ISO standards enhance patient safety?	Strongly agree	42.6	
	Agree	37.2	
	Neutral	12.1	
	Disagree	5.4	
	Strongly disagree	2.7	
	Total agreement	79.8	
2. Do ISO standards ensure the accuracy and calibration of medical devices?	Strongly agree	45.1	
	Agree	35.9	
	Neutral	11.2	
	Disagree	5.8	
	Strongly disagree	2.0	
	Total agreement	81.0	
3. Do ISO standards support clinical testing and risk management?	Strongly agree	40.3	
	Agree	37.7	
	Neutral	13.4	
	Disagree	6.8	
	Strongly disagree	1.8	
	Total agreement	78.0	
4. Are ISO standards effectively implemented in nonclinical settings?	Strongly agree	22.0	
	Agree	41.4	
	Neutral	21.3	
	Disagree	12.0	
	Strongly disagree	3.3	
	Total agreement	63.4	
5. Should more training be provided to enhance the understanding of ISO standards?	Strongly agree	48.0	
	Agree	35.0	
	Neutral	9.5	
	Disagree	5.0	
	Strongly disagree	2.5	
	Total agreement	83.0	
6. Do you agree that ISO standards enhance transparency in healthcare systems?	Strongly agree	41.0	
	Agree	37.6	
	Neutral	12.0	
	Disagree	6.2	
	Strongly disagree	3.2	
	Total agreement	78.6	
7. Do you agree that ISO standards improve healthcare service accessibility?	Strongly agree		38.5
	Agree		40.3
	Neutral		13.0
	Disagree		5.7
	Strongly disagree		2.5
	Total agreement		78.8
8. Do you agree that ISO standards help protect patient data?	Strongly agree		44.2
	Agree		35.4
	Neutral		11.2
	Disagree		6.3
	Strongly disagree		2.9
	Total agreement		79.6
9. Do you agree that ISO standards enhance the quality of healthcare services?	Strongly agree		36.8
	Agree		39.7
	Neutral		14.5
	Disagree		6.0
	Strongly disagree		3.0
	Total agreement		76.5
10. Do you agree that ISO standards help streamline healthcare management systems?	Strongly Agree		35.2
	Agree		41.0
	Neutral		13.1
	Disagree		7.2
11. Do you agree that ISO standards reduce the risk of medical errors?	Strongly agree		29.6
	Agree		43.5
	Neutral		15.4
	Disagree		8.5
	Strongly disagree		3.0
	Total agreement		73.1
12. Do you agree that ISO standards facilitate cross-border healthcare delivery?	Strongly agree		27.3
	Agree		38.9
	Neutral		19.1
	Disagree		9.7
	Strongly disagree		5.0
	Total agreement		66.2
13. Do you agree that ISO standards are critical for the adoption of new healthcare technologies?	Strongly agree		31.2
	Agree		42.8
	Neutral		16.5
	Disagree		6.7
	Strongly disagree		2.8
	Total agreement		74.0
14. Do you agree that ISO standards help healthcare institutions meet regulatory requirements?	Strongly agree		34.5
	Agree		43.1
	Neutral		13.8
	Disagree		6.1
	Strongly disagree		2.5
	Total agreement		77.6

ISO = International Organization for Standardization.

The age distribution analysis showed that professionals in their early 30s were more likely to recognize the benefits of ISO standards (*P* < .05). Younger professionals, particularly those working in advanced healthcare systems in urban regions, demonstrated greater familiarity and acceptance of ISO standards Table 4. The correlation between years of experience and perception of ISO standards was also significant (*P* < .05), with more experienced professionals showing higher recognition of ISO standards’ importance.

**Table 4:** Agreement on ISO standards by years of experience.

Years of experience	% agreement on ISO standards
1–5 yr	67.2
5–10 yr	74.5
10–15 yr	81.3
15+ yr	85.9

ISO = International Organization for Standardization.

**Figure 1.**
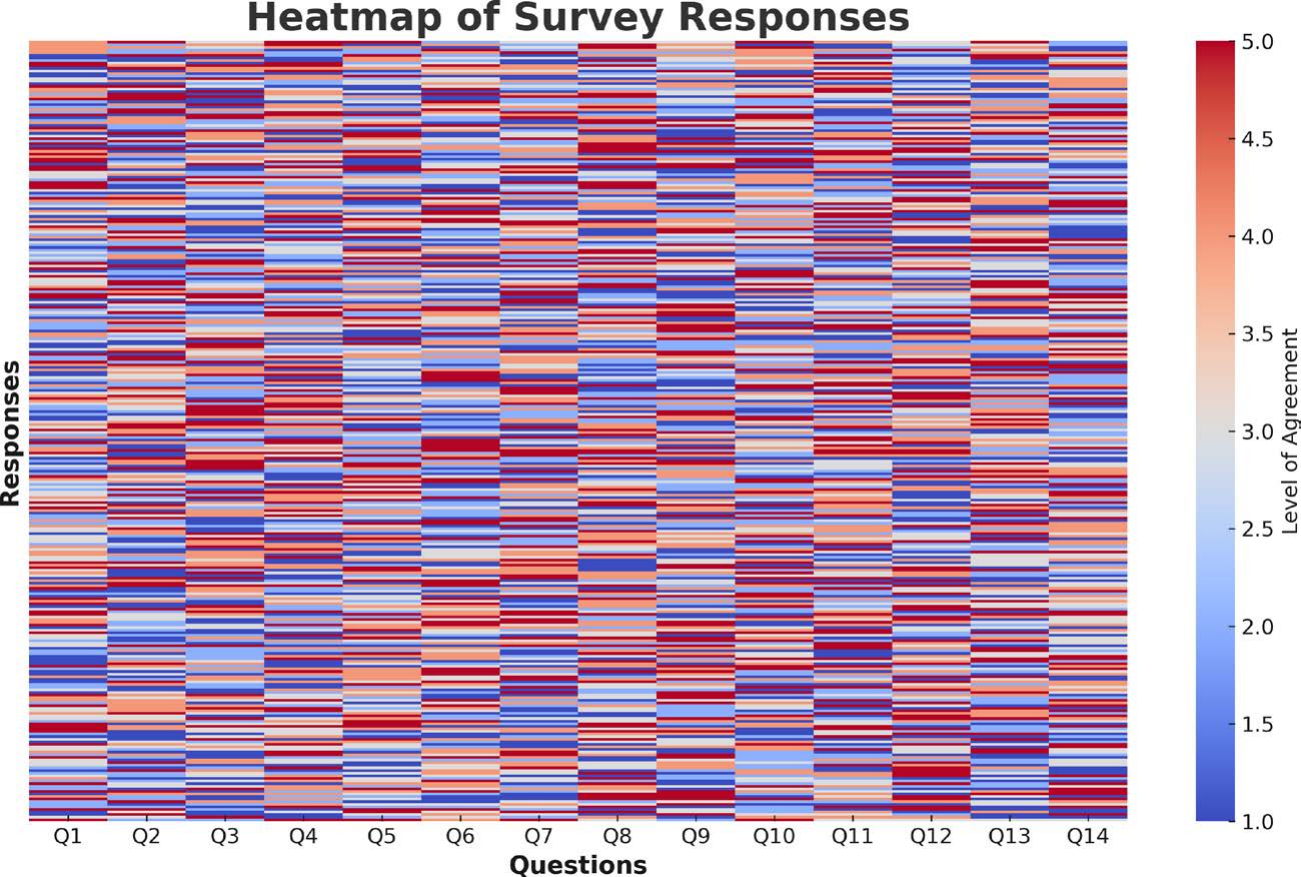
Heatmap of survey responses.

Figure 1 heatmap visualizes survey responses, identifying patterns across 14 questions, allowing quick identification of consensus and divergence, and identifying areas requiring further investigation.

## 4. Discussion

The survey results assess healthcare professionals in different areas of Saudi Arabia on their understanding and perceptions of ISO standards for patient safety, healthcare practices, and medical devices. The feedback from professionals of diverse backgrounds, including biomedical engineers, physicians (clinical and diagnostic), nurses, and healthcare administrators reflect the extent to which ISO standards have been achieved in the current Saudi healthcare setting and highlight major points for further improvement across various sectors of health service implementation. There was strong agreement on patient safety from across-the-board regions (Table 3; positive impact of ISO standards: agree, 79.8%). This strong view reflects international research showing that ISO standards are key to the standardization of health care delivery and in mitigating risks for medical errors.^[1,2]^ This high rate of agreement reflects the greater value given to ISO standards as tools to improve patient safety and quality of care in Saudi Arabia, a country that is experiencing significant progress in its healthcare sector.^[3]^ The importance of ISO standards in terms of calibration and accuracy of medical devices was universally agreed upon by 81.0% of participants.

Biomedical engineers, who constituted the largest professional group (43.3%) (Table 5), showed the highest level of agreement (89.3%), further underscoring the relevance of these standards in maintaining the reliability of healthcare technologies.^[5]^ As Saudi Arabia continues to expand its healthcare infrastructure, particularly in urban centers such as Riyadh, Jeddah, and the Eastern Province, ensuring consistent adherence to ISO standards for medical devices will be crucial in maintaining high-quality care across the country.^[4]^

**Table 5:** Distribution of respondents by profession.

Profession	Count
Biomedical engineer	143
Physician	87
Nurse	10
Health care administrator	9
Physician assistant	9
Other professions	17

While clinical adoption of ISO standards appears robust, the survey revealed significant gaps in nonclinical settings. Only 63.4% of respondents agreed that ISO standards were effectively implemented in nonclinical roles (Table 4), suggesting that administrative and support functions lag in terms of ISO compliance. Healthcare administrators (60.5%) and nurses (68.1%) displayed lower levels of agreement compared to biomedical engineers and physicians, highlighting a need for further education and training on the relevance of ISO standards beyond clinical practices.^[7]^

Regional differences in ISO standard perception were also noted, with respondents from Riyadh (81.5%) and Makkah (78.9%) showing stronger agreement than those from Asir (58.9%) and the Northern Borders (53.2%) (Table 2).

Difference in the structure of healthcare could be a factor, as more developed urban areas may wake up with slightly better resources and training than rural parts.^[9]^ As Saudi Arabia takes further strides toward Vision 2030, which aspires to advance healthcare provision at the national level, regional disparities must be addressed in modernizing its healthcare services toward enhanced access and quality for all regions.^[3]^ This average was one of a handful, which signals that more training in ISO standards is required – 83.0% of our respondents agreed with me on this point. These results are in concordance with previous studies highlighting the importance of education for optimal understanding and usage of ISO standards in healthcare surroundings as well.^[8]^ Training in particular is required, and perhaps especially so for rural regions where resources are less abundant and personnel less experienced. A heatmap in Figure 1 shows agreement and disagreement in answers to each of the 14 survey questions, revealing patterns within the data. There is broad agreement, for example, that ISO standards play an important role in improving patient safety, medical device performance, and clinical risk control; less consensus on their value in nonclinical use and training programs. This visualization shows the clear path to improve ISO adoption in the future.

The relationship between experience and perception of ISO standards was also examined. More experienced professionals, particularly those with over 10 years of experience, demonstrated higher levels of agreement regarding the importance of these standards (Table 4).

This implies that there is a lot of learning involved in understanding the importance of these standards, particularly at the leader and decision-maker level in healthcare organizations. Even the next generation of healthcare professionals, in their early 30s, agree to a high degree, which suggests that more healthcare professionals are being trained in ISO practices, particularly in specialized urban hospitals.^[13]^ The ANOVA analysis indicates a strong, significant association (*P* < 2 × 10^−16^) between age and years of professional experience, indicating that knowledge/implementation of ISO standards is more vital as healthcare professionals get older. ISO standards, best used to move organizations toward compliance and ongoing improvement by those with the most leadership or experience.

Although the perception of ISO standards is widely positive, there are still challenges to translating them into practice in nonclinical fields. The participation of the rural respondents and those in the administrative role showed less agreement; hence, more intentional interventions are still needed to foster the adoption of ISO standards in these areas.^[14]^ This gap is consistent with global findings on integrating ISO standards into nonclinical operations, especially in areas where healthcare infrastructures are not uniform.^[10]^

## 5. Conclusion

The findings of this survey give an insight into the perception of ISO standards in the healthcare sector within Saudi Arabia. Many respondents agreed on the importance of ISO standards in clinical practice, particularly the positive impact of these standards on patient safety and the accuracy of medical devices.

Nevertheless, there are gaps remaining in nonclinical adoption, due largely to awareness, training, and resources, which must be filled. These factors should be addressed as barriers to the implementation of ISO standards, and future efforts should concentrate on this goal, especially in rural and nonclinical sectors, which ultimately serve the mandate for standardized provision of healthcare across the Saudi healthcare landscape.

More research regarding the challenges faced in nonclinical roles and rural regions should be encouraged, with an emphasis on specialized training programs as well as infrastructural development to adopt a healthcare system that is according to ISO standards and in line with international best practices and Vision 2030’s ambitious goals.

## Acknowledgments

The authors, therefore, acknowledge with thanks WAQF and the Deanship of Scientific Research (DSR) for technical and financial support.

## Author contributions

**Funding acquisition:** Eyad Talal Attar.

**Investigation:** Eyad Talal Attar.

**Methodology:** Eyad Talal Attar.

**Project administration:** Eyad Talal Attar.

**Software:** Eyad Talal Attar.

**Supervision:** Eyad Talal Attar.

**Writing – original draft:** Eyad Talal Attar.
